# Self-reported cognitive and psychiatric symptoms at 3 months predict single-item measures of fatigue and daytime sleep 12 months after ischemic stroke

**DOI:** 10.3389/fneur.2022.944586

**Published:** 2022-11-17

**Authors:** Elisabeth Kliem, Angela Susan Labberton, Mathias Barra, Alexander Olsen, Bente Thommessen, Owen Thomas, Elise Gjestad, Bent Indredavik, Ramune Grambaite

**Affiliations:** ^1^Department of Psychology, Norwegian University of Science and Technology, Trondheim, Norway; ^2^The Health Services Research Unit – HØKH, Akershus University Hospital HF, Lørenskog, Norway; ^3^Division for Health Services, Norwegian Institute of Public Health, Oslo, Norway; ^4^Bergen Center for Ethics and Priority Settings (BCEPS), University of Bergen, Bergen, Norway; ^5^Department of Physical Medicine and Rehabilitation, St. Olav's Hospital, Trondheim University Hospital, Trondheim, Norway; ^6^Department of Neurology, Akershus University Hospital, Lørenskog, Norway; ^7^Clinic of Medicine, St. Olavs Hospital, Trondheim University Hospital, Trondheim, Norway; ^8^Stroke Unit, Department of Medicine, St. Olav's Hospital, Trondheim University Hospital, Trondheim, Norway; ^9^Department of Neuromedicine and Movement Science, Faculty of Medicine and Health Science, Norwegian University of Science and Technology, Trondheim, Norway

**Keywords:** memory, depression, anxiety, subjective, cognition, vascular disease, concentration, daytime function

## Abstract

**Introduction:**

Post-stroke fatigue and increased need for daytime sleep are multidimensional and insufficiently understood sequelae. Our aim was to study the relationships of self-reported cognitive and psychiatric symptoms at 3 months with fatigue and daytime sleep at 12 months post-stroke.

**Methods:**

Ischemic stroke patients without reported history of dementia or depression completed postal surveys 3- and 12-months post-stroke. At 3 months, psychiatric symptoms were assessed with the Hospital Anxiety and Depression Scale (HADS), and self-reported changes in cognitive symptoms (concentration and memory) compared to pre-stroke were assessed using single-item measures. At 12 months, single-item questions about changes in self-reported difficulties sleeping at night, fatigue and daytime sleep were included. First, we studied whether self-reported cognitive and/or psychiatric symptoms at 3 months were associated with daytime sleep and fatigue at 12 months using multiple logistic regression. Second, we fitted 2 structural equation models (SEMs) predicting fatigue and 2 models predicting daytime sleep. We compared a model where only age, sex, stroke severity (National Institutes of Health Stroke Scale; NIHSS), and difficulties sleeping at night predicted fatigue and daytime sleep at 12 months to a model where mental distress (i.e., a latent variable built of cognitive and psychiatric symptoms) was included as an additional predictor of fatigue and daytime sleep at 12 months.

**Results:**

Of 156 patients (NIHSS within 24 hours after admission (*mean* ± *SD*) = 3.6 ± 4.3, age = 73.0 ± 10.8, 41% female) 37.9% reported increased daytime sleep and 50.0% fatigue at 12 months. Increased psychiatric symptoms and worsened cognitive symptoms were associated with fatigue and daytime sleep at 12 months, after controlling for NIHSS, age, sex, and difficulties sleeping at night. SEM models including mental distress as predictor showed adequate model fit across 3 fit measures (highest RMSEA = 0.063, lowest CFI and TLI, both 0.975). Models without mental distress were not supported.

**Conclusion:**

Self-reported cognitive and psychiatric symptoms at 3 months predict increased daytime sleep and fatigue at 12 months. This highlights the relevance of monitoring cognitive and psychiatric symptoms in the subacute phase post-stroke. However, future research using validated measures of self-reported symptoms are needed to further explore these relationships.

## Introduction

Every 40 s someone suffers from a stroke worldwide (1), and more than 80 million people are living with stroke sequelae (2). For stroke survivors, appropriate functioning during daytime is crucial to successfully return to work and to increase independence in activities of daily living after stroke (3, 4).

Post-stroke fatigue majorly impairs patients' daytime functioning, and is commonly reported as one of the most burdensome symptoms following stroke (5). It substantially impedes patients' quality of life after stroke (6) and is associated with higher institutionalization and mortality (7).

Fatigue is a complex condition which has been described as the feeling of disproportionate physical or psychological exhaustion and lack of energy even when engaging in simple activities (8). About half of all patients report fatigue at some point after stroke (9), although prevalence rates vary greatly depending on the methodology and assessment tools used. Post-stroke fatigue appears to be rather persistent from the acute phase to at least 3 years after stroke (10), and understanding which factors contribute to fatigue may lead to advances in developing more appropriate and effective intervention strategies.

Our understanding of post-stroke fatigue, including its possible biological etiology, remains limited (11, 12). Fatigue after stroke may be related to the severity of neurological impairment (13) and neurophysiological changes due to the damage of the brain tissue (14). Other factors have been suggested as possible contributors to post-stroke fatigue, such as sleep problems (12), pain, other comorbidities and medication use (10). In addition, women seem to be at a higher risk for post-stroke fatigue (15), while the association between age and fatigue remains somewhat unclear (12).

Two key factors that are likely to be relevant for the development of post-stroke fatigue are cognitive and psychiatric symptoms. Cognitive symptoms such as memory or concentration problems are commonly reported early after stroke (16) and remain relatively stable from 3 to 12 months (17). Similarly, post-stroke psychiatric symptoms are common, with as many as 1 in 3 patients experiencing depression (18) and 1 in 4 reporting anxiety during the first-year post-stroke (19). Different mechanisms may contribute to self-reported cognitive and psychiatric symptoms predicting fatigue post-stroke: For example, cognitive symptoms may require a patient to use more effort when fulfilling simple everyday tasks which, over time, may trigger fatigue (20). Additionally, symptoms of depression are commonly associated with sleep problems and decreased physical activity, both of which may result in fatigue (12), and also be a perpetuating factor of fatigue (11). Examining temporal associations between these symptoms after stroke may therefore provide valuable knowledge on the etiology of post-stroke fatigue.

However, studying associations between psychiatric symptoms and fatigue is challenging due to the subjective nature of, and overlap between, these symptoms. After stroke, fatigue and psychiatric symptoms may occur independently of each other (13), but they often co-occur, with one third of stroke survivors experiencing both (21, 22). This is also reflected in fatigue being included in the diagnostic criteria of depression in the 5th edition of the Diagnostic and Statistical Manual of Mental Disorders (DSM-5) (23). Symptoms of depression and anxiety may also play a role in developing fatigue in the long-term post-stroke (5, 13), but research is sparse (10).

Similarly, temporal relationships between subacute cognitive symptoms and fatigue in the long-term remain poorly understood. Cross-sectional findings indicate an association of decreased performance-based learning, memory, processing speed and attention with fatigue after stroke (24), but findings on the relationship between *self-reported* cognitive symptoms and fatigue are inconclusive (25): Whereas some findings indicate that self-reported mental slowness may be associated with fatigue post-stroke (26), other studies did not find a clear relationship between self-reported cognitive symptoms and fatigue after ischemic stroke when controlling for depressive symptoms (27).

Besides fatigue, sleeping during daytime is common in stroke patients (28), and may further impair daytime functioning and engagement in rehabilitation after stroke. Interestingly, despite its possibly deteriorating effects on recovery, research on increased daytime sleep after stroke is sparse, and has mostly focused on daytime *sleepiness*, i.e. a person's tendency to fall asleep (29). Even though daytime sleepiness, daytime sleep, and fatigue are related constructs which may overlap (30), they could have different pathogenesis, making differentiating between these constructs important. Yet, little is known about daytime *sleep* among stroke patients in general, and about its relation to cognitive and psychiatric symptoms in particular. Some results indicate that sleep during daytime is related to depression in the general population, independent from life style factors, use of sleep medicine, and comorbidities (31). However, findings on the relationship between cognitive symptoms and daytime sleep are inconclusive (32). For example, some longitudinal findings suggest that daytime sleep may predict cognitive decline (33), whereas others found a protective effect of daytime sleep for cognitive function (34). Different factors such as duration (32), frequency, and intentionality (35) of daytime sleep may explain these inconsistent findings, but whether specific daytime sleep patterns may have differential effects on cognitive symptoms still remains unclear (32). So far, research has focused on studying whether daytime sleep predicts cognitive symptoms. Studies on the possible predictive effect of cognitive and psychiatric symptoms for daytime sleep are, however, lacking. Also, most studies have focused on performance-based cognitive function, and little is known about the role of self-reported cognitive symptoms in predicting daytime sleep in general, and in the stroke population specifically.

Both daytime sleep and fatigue are multidimensional stroke sequelae, and their development may be best predicted by a complex interplay between various factors. In clinical practice it is typically difficult to distinguish self-reported psychiatric and cognitive symptoms from each other, and patients reporting these symptoms may experience such problems as a more general feeling of being somewhat different compared to before the stroke (36). Such self-reported symptoms may therefore reflect increased mental distress in these patients, and studying the role of self-reported mental distress in predicting fatigue and daytime sleep post-stroke may help to increase our understanding of the etiology of these stroke sequelae. However, we lack studies that specifically address the complex nature of daytime sleep and fatigue by taking various factors, including mental distress, and their interactions over time into account.

Using a longitudinal study design and self-report measures, the objective of this study was to investigate temporal relationships of self-reported symptoms with fatigue and daytime sleep, while also taking the complex, multidimensional nature of both daytime sleep and fatigue into account. Therefore, we (1) studied whether self-reported cognitive and/or psychiatric symptoms 3 months after ischemic stroke were associated with increased self-reported fatigue after 12 months using multiple regression. We hypothesized that self-reported decline in memory and concentration as well as higher levels of anxiety and depression would predict increased fatigue in the long-term. As little is known about possible predictors of daytime sleep post-stroke, we (2) fitted multiple regression models to explore whether self-reported cognitive and psychiatric symptoms at 3 months were associated with increased daytime sleep at 12 months. Finally, to account for the complex interplay between various factors in predicting daytime sleep and fatigue after stroke, we (3) used structural equation modeling (SEM) to further study the predictive value of self-reported mental distress for fatigue and daytime sleep at 12 months (see Figures 2–5).

## Methods

### Study design and subjects

This study used the NORSPOT data (Norwegian Stroke—Paths of Treatment) which were collected at the Stroke Unit (SU) at Akershus University hospital (Ahus) in Norway. The Ahus SU is classified as a comprehensive stroke center according to European Stroke Organization (ESO) standards.

All patients admitted to the SU during the period from 15th February 2012 to 15th March 2013 were included in the NORSPOT cohort. Patients were classified by experienced neurologists based on clinical symptoms and supported by imaging data (computed tomography or magnetic resonance imaging) using the following International Classification of Diseases-10th revision (ICD-10) codes: ischemic stroke (I63.X), intracerebral hemorrhage (I61.X) and transient ischemic attack (TIA; G45.X, excluding G45.4). In total, 1,189 unique patients with either TIA or stroke were recorded, who were eligible for postal survey questionnaires 3 and 12 months after discharge from the SU.

**Figure 1:**
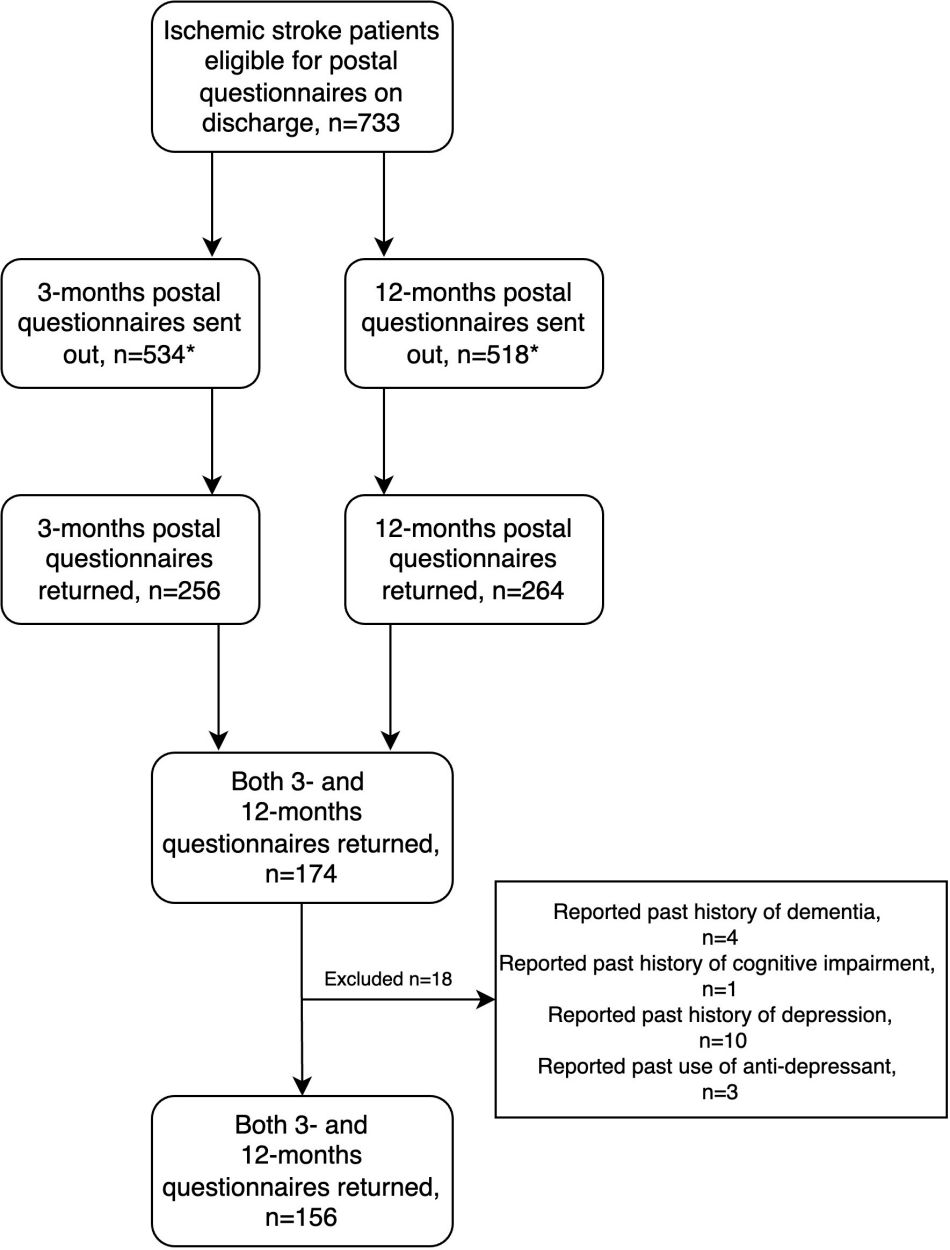
*n < 733 because of death, missing address data and late inclusion.

For the present study, only patients with cerebral infarctions who returned both the 3- and 12-month questionnaire were included. In addition, based on hospital records and self-report at admission, patients with a known past history of either depression (including known use of antidepressants), cognitive impairment, or dementia were excluded (see Figure 1 for the inclusion flowchart). The NORSPOT protocol, including information about the follow-up questionnaires, is described in more detail in an earlier publication (37).

### Ethics

The NORSPOT project was considered to be a quality assurance project by the Regional Committee for Medical and Health Research Ethics and approval was granted by the Data Protection Officer at Akershus University Hospital (ref. no. 11–076). Consent to participate was not required due to the nature of the studies (retrospective chart audit and quality assurance, respectively). In addition, for the follow-up questionnaires, each recipient was informed about the NORSPOT project in a cover letter and asked to return the questionnaire only if they agreed for their data to be used for statistical analyses.

### Measures

All data used in this study were collected *via* the study's approved hospital forms and follow-up postal questionnaires.

#### Self-reported psychiatric symptoms

Self-reported psychiatric symptoms were assessed using the Hospital Anxiety and Depression Scale (HADS) (38) 3 months after hospital discharge. The HADS consists of 2 subscales evaluating depression (HADS-D) and anxiety (HADS-A) with 7 items each. HADS does not include any questions related to fatigue or sleep. The questionnaire has been validated in stroke patients in general (39), and the Norwegian version has shown to be a valid screening tool in a Norwegian stroke population (40). All items are rated on a four-point scale from ‘not at all' (0) to ‘most of the time' (3). A score ≥8 on either subscale indicates possible clinically relevant symptoms (41). In cases where one item was missing on a subscale, the mean of the 6 completed items was imputed in accord with recommended procedure for analyses relying on summary statistics (e.g., sample means) (42). Cases with more than 1 missing item on a subscale were excluded from further analyses.

#### Self-reported cognitive symptoms, daytime sleep, fatigue and difficulties sleeping at night

Self-reported memory and concentration were assessed with one item each at the 3-month follow-up. Patients were asked to indicate whether they experienced changes in their concentration and memory at the time of assessment compared to before their stroke by rating their memory and concentration as either “worsened”, “unchanged” or “improved”.

Daytime sleep, difficulties sleeping at night and fatigue were all assessed with one self-report item at the 12-month follow-up. Patients were asked to rate their difficulties sleeping at night and general weariness/fatigue at the time of assessment compared to before stroke as either “worsened”, “unchanged” or “improved”. Self-reported daytime sleep was assessed by asking “*Do you sleep more during the day than before admission to the hospital?”* with “yes”, “no”, and “unsure” as answer categories. For all these items, the patients were specifically asked to think of the index admission to the SU (in case of readmissions) 3 and 12 months prior, respectively.

#### Stroke severity

The National Institutes of Health Stroke Scale (NIHSS) was used to assess stroke severity by quantifying neurologic impairment within 24 h after admission to the hospital. Where a prospective NIHSS score (either at admission or within 24 h after admission) was unavailable, a NIHSS score was assigned retrospectively by the second author (ASL) using admission records and a validated algorithm (43). The NIHSS is composed of 11 items, each scoring a specific ability, e.g., language or motor function. Item scores range from 0 (= normal function), to 1 to 4 (= different levels of impairment). The highest possible score for non-comatose patients is 42.

### Statistical analyses

#### Descriptive statistics and regression analyses

The Statistical Package for Social Sciences, version 25 (44), was used. To compare self-reported cognitive symptoms in patients with and without clinical symptoms of depression and anxiety (HADS) χ^2^ tests were used. Associations between self-reported fatigue and daytime sleep were studied using Spearman correlation, and Pearson correlation was used for continuous variables. These analyses were conducted to get a better understanding of our sample as a whole, as well as to get an indication of whether the self-reported measures used in this study might load on a common factor. To examine whether the analyzed sample significantly differed from those patients without 3- and 12-months data in any of the key baseline variables, χ^2^ tests were used for categorical variables, and independent samples *t*-tests were used for continuous variables.

To estimate effect sizes for categorical variables, Cramer's phi (ϕ_c_) was used with ϕ_c_ = 0.10 indicating a small, ϕ_c_ = 0.30 a medium, and ϕ_c_ = 0.50 a large effect when the number of degrees of freedom = 1 (45). For continuous variables, Cohen's d was used, where d ≥ 0.20 indicates a small, d ≥ 0.50 a medium, and d ≥ 0.80 a large effect (45).

The relationships of self-reported psychiatric and cognitive symptoms with daytime sleep and fatigue were analyzed using logistic regressions. All assumptions for binary and ordinal logistic regression, including the assumption of proportional odds, were met. As for daytime sleep, we assumed that the answer category ‘*unsure*' includes patients who may sleep more during the day on some days (but not on other days), thereby reflecting a medium degree of daytime sleep. We therefore assumed that the ‘*unsure'* category can be treated as an intermediate category between “Yes, I do sleep more during the day” and “No, I do not sleep more during the day”; thus, the 3 answer categories can be naturally ordered by the degree of self-reported increase in daytime sleep (“no” > “unsure” > “yes”) with no metric for their “distance”. Self-reported daytime sleep was therefore treated as an ordinal outcome variable. Due to low frequencies of improved self-reported symptoms (≤ 5%), these variables were recoded as binary variables in the regression models with 0 = “unchanged or improved” and 1 = “worsened”. Consequently, binary logistic regression was used in primary regression analyses with fatigue as the dependent variable, with the following specifications:


(1) $$\begin{array}{l} Fatigue=\beta_{0}X+\beta_{1}age+\beta_{2}sex+\beta_{3}NIHSS\\ +\beta_{4}Difficulties\;sleeping\;at\;night \end{array}$$

where X was either HADS-A, HADS-D, self-reported memory, or concentration. In each of these analyses we were only interested in testing the significance of the coefficient (β_0_) of *X*, and therefore imposed a Bonferroni correction of *p* ≤ 0.0125 (i.e., *p* ≤ 0.05/4). For exploratory regression analyses with daytime sleep as the dependent variable, we fitted ordinal logistic regression models with the equivalent model specifications as listed above. Based on existing literature, age, sex, NIHSS and difficulties sleeping at night were included as control variables.

#### Structural equation modeling analyses

Structural equation modeling (SEM) is a hybrid technique that combines aspects of path analysis, regression and confirmatory factor analysis and allows for testing of how well a prescribed theoretical model fits the given data. Thus, it allows for the evaluation of a priori specified relations between variables, and for the comparison between competing theoretical models specified by the researcher (46). All SEM analyses were conducted using the *lavaan*, package version 0.6-10 (47) with the R Statistical Computing Software version 3.6.1 (48).

In our models, we included both observed (i.e., directly measured variables that are described under “Method”) and a latent variable (i.e., unobserved, theoretically built construct). Specifically, as increased psychiatric and cognitive symptoms may reflect increased mental distress in these patients, we built a latent variable of *mental distress* based on the observed variables HADS-A and -D, and self-reported concentration and memory at 3 months. Significant regressor scores (*p* ≤ 0.05) of these observed variables confirmed their contribution to the latent variable (confirmatory factor analysis), and the default marker method which fixes the factor loading of the first indicator (HADS-A) to 1 was used. All observed variables were standardized before entering the analysis. As our endogenous variables were categorical/ordinal, diagonally weighted least squares with robust standard errors (DWLS, estimator: WLSM) was used to estimate the model parameters and the full weight matrix to compute a mean-adjusted test statistic. Missing data were handled by listwise deletion.

The following fit indices were used to assess model goodness-of-fit: the χ^2^ statistic, the robust comparative fit index (robust CFI), the robust Tucker–Lewis index (robust TLI), and the robust root mean square error approximation (robust RMSEA). Thus, following recommendations (49), we used both incremental fit indices (CFI, TLI) which compare the fit of a theoretical model relative to a null model where all observed variables are uncorrelated, and an absolute fit index (RMSEA) which assesses how well the specified theoretical model reproduces the covariance matrix observed, in addition to the χ^2^ statistic.

Hu and Bentler (50) cut-off scores were used where RMSEA values ≤ 0.06 and CFI/TLI ≥ 0.95 are considered relatively good model fit. χ^2^ difference test with Satorra and Bentler's (51) method for mean-adjusted test statistics was used to compare nested models.

**Figure 2:**
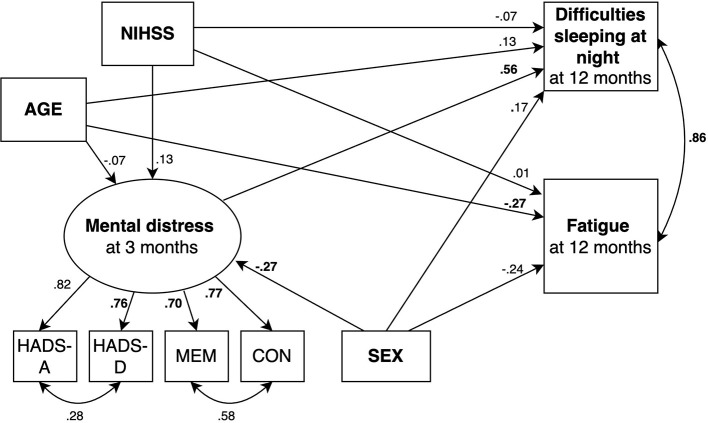
SEM model predicting fatigue where mental distress is not included as predictor. Circles depict latent (unobserved theoretically built) variables. Squares depict observed (measured) variables. Directional arrows indicate direct effects of one variable to another (regressor paths). Bidirectional arrows show covariances, and no directional association between two variables. Numbers indicate completely standardized (where both observed and latent variables are standardized) parameters. Bold numbers indicate significant parameters (*p* ≤ 0.05). The default marker method which fixes the factor loading of the first indicator to 1 was used for the latent variable. For ease of representation error terms are not shown in the figure. MEM, Self-reported memory; CON, Self-reported concentration.

**Figure 3:**
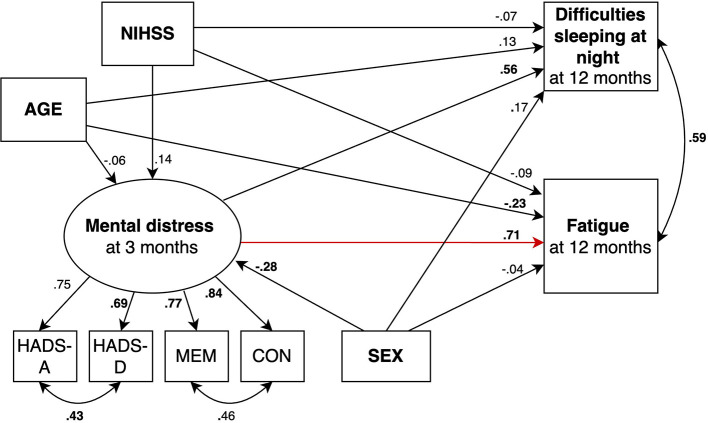
SEM model predicting fatigue where mental distress is included as predictor (red arrow). Circles depict latent (unobserved theoretically built) variables. Squares depict observed (measured) variables. Directional arrows indicate direct effects of one variable to another (regressor paths). Bidirectional arrows show covariances, and no directional association between two variables. Numbers indicate completely standardized (where both observed and latent variables are standardized) parameters. Bold numbers indicate significant parameters (*p* ≤ 0.05). The default marker method which fixes the factor loading of the first indicator to 1 was used for the latent variable. For ease of representation error terms are not shown in the figure. MEM, Self-reported memory; CON, Self-reported concentration.

**Figure 4:**
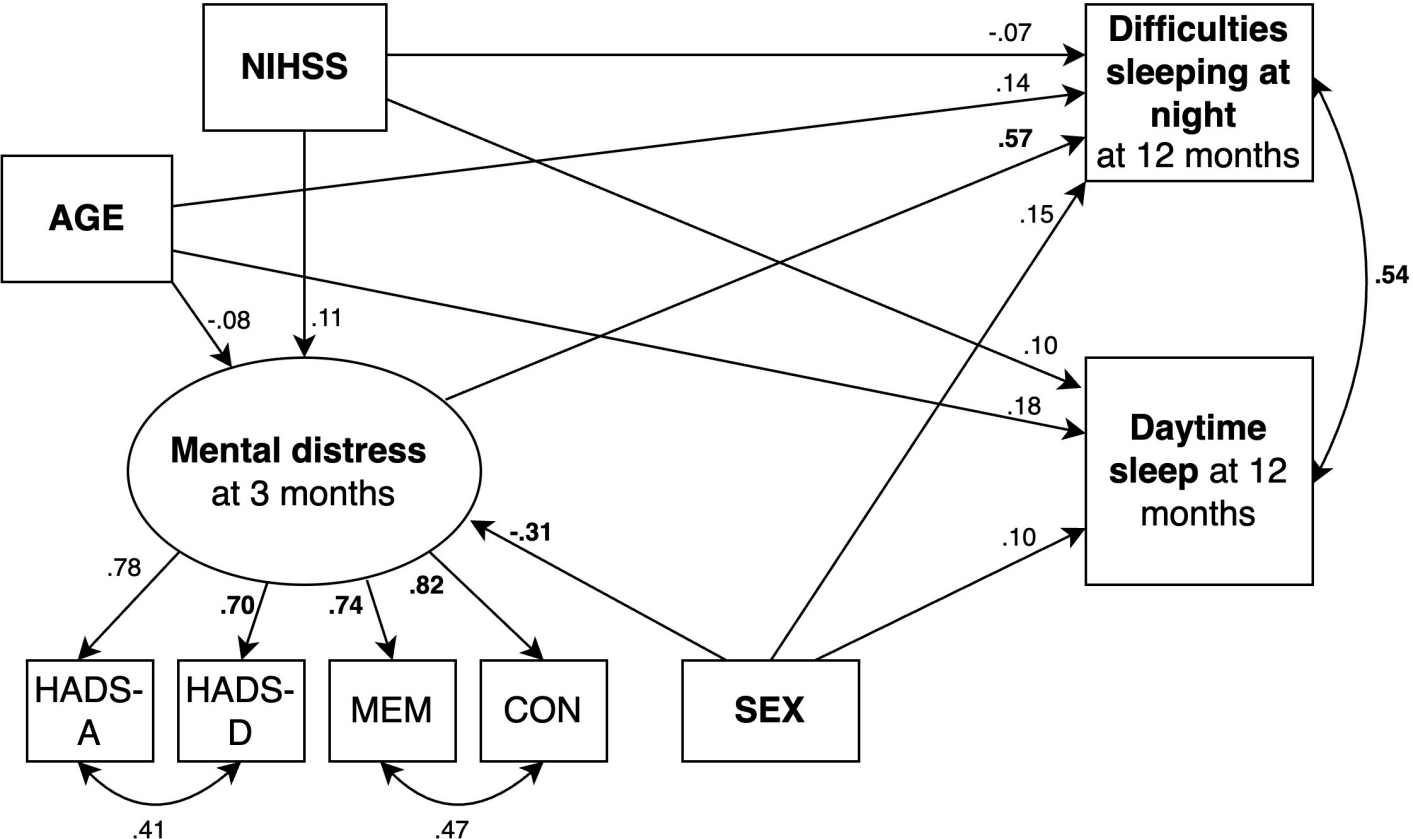
SEM model predicting daytime sleep where mental distress is not included as predictor. Circles depict latent (unobserved theoretically built) variables. Squares depict observed (measured) variables. Directional arrows indicate direct effects of one variable to another (regressor paths). Bidirectional arrows show covariances, and no directional association between two variables. Numbers indicate completely standardized (where both observed and latent variables are standardized) parameters. Bold numbers indicate significant parameters (*p* ≤ 0.05). The default marker method which fixes the factor loading of the first indicator to 1 was used for the latent variable. For ease of representation error terms are not shown in the figure. MEM, Self-reported memory; CON, Self-reported concentration.

**Figure 5:**
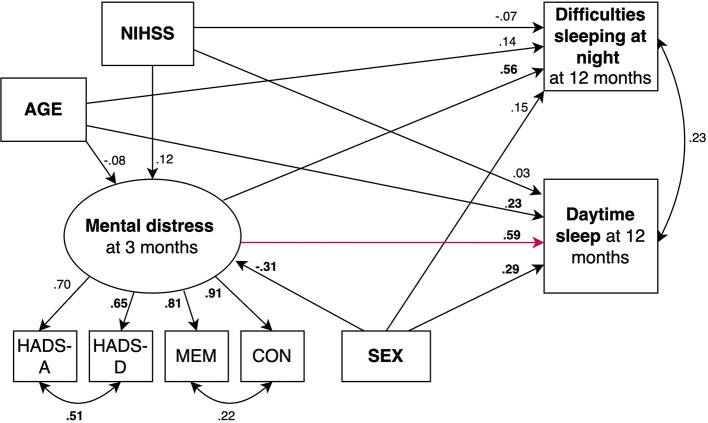
SEM model predicting daytime sleep where mental distress is included as predictor (red arrow). Circles depict latent (unobserved theoretically built) variables. Squares depict observed (measured) variables. Directional arrows indicate direct effects of one variable to another (regressor paths). Bidirectional arrows show covariances, and no directional association between two variables. Numbers indicate completely standardized (where both observed and latent variables are standardized) parameters. Bold numbers indicate significant parameters (*p* ≤ 0.05). The default marker method which fixes the factor loading of the first indicator to 1 was used for the latent variable. For ease of representation error terms are not shown in the figure. MEM, Self-reported memory; CON, Self-reported concentration.

We fitted 2 structural equation models predicting fatigue (Figures 2, 3) and 2 models predicting daytime sleep at 12 months (Figures 4, 5). Specifically, we compared a model where only age, sex, stroke severity (NIHSS), and difficulties sleeping at night predicted fatigue and daytime sleep at 12 months (Model 1 and Model 3, respectively) to a model where mental distress was included as additional predictor of fatigue and daytime sleep at 12 months (Model 2 and Model 4, respectively).

## Results

### Patient characteristics

In total, data from 156 patients who answered both the 3- and 12-month questionnaire and met the inclusion criteria were analyzed. Table 1 displays patient characteristics and Table 2 frequencies of self-reported symptoms. Only a few patients reported improved cognitive symptoms, with 2 patients (1.5%) reporting improved memory and concentration at 3 months. Two (1.4%) and 7 (5.1%) patients reported improved fatigue and improved nighttime sleep at 12 months, respectively. Fifteen patients (9.8%) answered “unsure” to the question of whether they slept more during the day.

**Table 1:** Patient characteristics (*n* = 156).

**Domain**	**Value**	**Data available**
Age in years at inclusion, M (SD)	73.0 (10.8)	*n =* 156
Women, *n* (%)	64 (41.0%)	*n =* 156
Education		*n =* 132
*Primary school, n (%)*	26 (19.7%)	
*Secondary school, n (%)*	58 (43.9%)	
*3 years of university, n (%)*	27 (20.5%)	
*5 years of university, n (%)*	21 (15.9%)	
NIHSS score within 24 h after admission, M (SD)	3.59 (4.3)	*n =* 140
Previous cerebrovascular disease		*n =* 156
*Previous TIA, n (%)*	15 (9.6%)	
*Previous infarct, n (%)*	20 (12.8%)	
*Previous TIA and infarct, n (%)*	4 (2.6%)	
*Previous hemorrhage, n (%)*	0 (0%)	

M, mean; SD, standard deviation; NIHSS, NIH stroke scale score; max. score = 42. TIA, Transient ischemic attack.

**Table 2:** Frequencies of self-reported symptoms at 3 and 12 months.

**Domain**	**3 months, *n* (%)**	**Domain**	**12 months, *n* (%)**
HADS-A score ≥ 8	31/151 (20.5%)	Increased daytime sleep	58/153 (37.9%)
HADS-D score ≥ 8	30/147 (20.4%)	Increased fatigue	69/138 (50.0%)
Worsened concentration	42/135 (31.1%)	Increased difficulties sleeping at night	26/137 (19.0%)
Worsened memory	49/135 (36.3%)	N/A	

Five (3.2%) and 12 (7.7%) patients had 1 missing item on HADS-D and HADS-A at 3 months, respectively. For these patients, imputed values were used to determine HADS-D and HADS-A scores. In total, 9 (5.8%) patients had more than one item missing on one of the subscales and were excluded from further analyses.

To investigate if patients included in the analyses significantly differed from those ischemic stroke patients from the NORSPOT cohort that were not included, we compared key baseline characteristics of these 2 samples. The analyzed sample (patients who answered both the 3- and 12-month questionnaire and met the inclusion criteria; *n* = 156) had milder strokes and better general cognitive function compared to patients where data at 3- and/or 12-months was unavailable (*n* = 559) (Supplementary Table 1).

### Cross-sectional associations between self-reported symptoms

At 3 months, self-reported decline in memory was strongly correlated with decline in concentration (χ^2^ = 50.85, df = 1, ϕ_c_ = 0.63, *p* ≤ 0.001). Likewise, self-reported symptoms of anxiety were strongly positively associated with symptoms of depression at 3 months (r = 0.67, 99%CI (0.53, 0.77), *p* ≤ 0.001). There were moderate associations between self-reported cognitive and psychiatric symptoms, with the association of symptoms of anxiety and depression with self-reported concentration being somewhat stronger than their relationship with memory (Table 3).

**Table 3:** Associations between self-reported symptoms at 3 months.

**Cognitive symptoms at 3 months**	**Psychiatric symptoms at 3 months**					
	**HADS-D**		**χ^2^**	***df***	***p***	**ϕ_c_**
	**Above cut-off**	**Below cut-off**				
Worsened concentration, *n* (%)	20 (71.4%)	21 (20.2%)	27.95	1	<0.001	0.45
Worsened memory, *n* (%)	20 (74.1%)	28 (26.7%)	20.86	1	<0.001	0.40
	**HADS-A**		χ^2^	***df***	***p***	ϕ_c_
	**Above cut-off**	**Below cut-off**				
Worsened concentration, *n* (%)	20 (66.7%)	22 (21.2%)	22.41	1	<0.001	0.41
Worsened memory, *n* (%)	19 (65.6%)	30 (28.6%)	13.37	1	<0.001	0.32

A score ≥ 8 on either HADS-subscale was used as cut-off. Numbers in parentheses indicate how many percent of patients had worsened cognitive symptoms in a given group (above or below cut-off on HADS-A/D).

At 12 months, increased sleep during daytime was significantly associated with self-reported increased fatigue (r_s_ = 0.28, 99%CI (0.06, 0.42), *p* ≤ 0.001) and difficulties sleeping at night (r_s_ = 0.27, 99%CI (0.05, 0.47), *p* ≤ 0.001). Increased fatigue was moderately correlated with difficulties sleeping at night at 12 months (χ^2^ = 18.86, df = 1, ϕ_c_ = 0.38, *p* ≤ 0.001).

### Relationship of self-reported symptoms at 3 months with fatigue and daytime sleep at 12 months

#### Logistic regressions results

Results of the primary regression analyses with fatigue as the dependent variable are displayed in Table 4. Worsened self-reported concentration and memory as well as higher symptoms of anxiety and depression at 3 months were associated with increased fatigue at 12 months (*p* ≤ 0.0125). Also, difficulties sleeping at night and lower age were associated with increased fatigue in all multivariate regression models (*p* ≤ 0.0125). None of the other covariates were associated with fatigue in any of the models (Supplementary Tables 2–4).

**Table 4:** Relationship of self-reported symptoms at 3 months with fatigue at 12 months.

**Increased fatigue (12 months)**						
		**95% CI**				
**Self-reported symptoms (3 months)**	**OR**	**Lower**	**Upper**	***p***	***R** ^2^*	*χ^2^ **(p)***
HADS-A-model	1.28	1.12	1.47	<0.001^*^	0.43	43.72 (<0.001)
HADS-D-model	1.22	1.08	1.37	0.001^*^	0.39	37.89 (<0.001)
Worse concentration-model	7.68	2.35	25.04	<0.001^*^	0.42	38.86 (<0.001)
Worse memory-model	4.05	1.48	11.10	0.007^*^	0.40	37.13 (<0.001)

* Significant result at p ≤ 0.0125. R^2^ = Nagelkerke R^2^. Four regression models were fitted, each containing one of the predictors of interest (self-reported symptoms), in addition to the four covariates age, sex, NIHSS and difficulties sleeping at night at 12 months. OR, CI, and p refer only to the predictor of interest, R^2^ indicates the amount of variance explained by the whole model, i.e. with all predictors included. χ^2^ = Omnibus test for model coefficients.

Results of the secondary regression analyses with daytime sleep as dependent variable are displayed in Table 5. Self-reported worsened cognitive symptoms and increased psychiatric symptoms were associated with increased daytime sleep. Of the included covariates, only increased difficulties sleeping at night were associated with a higher risk for increased daytime sleep at 12 months in all models (*p* ≤ 0.05; Supplementary Tables 5–9).

**Table 5:** Relationship of self-reported symptoms at 3 months with daytime sleep at 12 months.

**Increased daytime sleep (12 months)**					
		**95% CI**			
**Self-reported symptoms (3 months)**	**OR**	**Lower**	**Upper**	***R** ^2^*	*χ^2^ **(p)***
HADS-A-model	1.11	1.01	1.24	0.13	12.97 (0.024)
HADS-D-model	1.11	1.01	1.22	0.13	13.10 (0.022)
Worse concentration-model	4.89	1.85	12.93	0.22	21.96 (<0.001)
Worse memory-model	3.39	1.45	7.93	0.19	18.14 (0.003)

R^2^ = Nagelkerke R^2^. Four regression models were fitted, each containing one of the predictors of interest (self-reported symptoms), in addition to the four covariates age, sex, NIHSS and difficulties sleeping at night at 12 months. OR, CI, and p refer only to the predictor of interest, R^2^ indicates the amount of variance explained by the whole model, i.e. with all predictors included. χ^2^ = Omnibus test for model coefficients.

#### SEM results

Beta-coefficients for each parameter are shown in Figures 2–5. The 2 models including mental distress as predictor of fatigue and daytime sleep at 12 months (Model 2 and Model 4, respectively) showed adequate fit to the data across 3 fit measures (highest RMSEA = 0.063, lowest CFI and TLI, both 0.975). The models where mental distress was not included (Model 1 and Model 3) were not supported by the data (see Table 6). χ^2^ difference tests comparing nested models indicated that the models including mental distress performed significantly better than those models without the latent variable included when predicting fatigue (χ^2^_difference_ = 20.80, df_difference_ = 1, p_difference_ = < 0.001) and daytime sleep (χ^2^_difference_ = 17.08, df _difference_ = 1, p_difference_ = < 0.001).

**Table 6:** Goodness-of-fit indices for all SEM models.

						**90% CI**			
**SEM**		**χ^2^**	**df**	***p***	**RSMEA**	**Lower**	**Upper**	**TLI**	**CFI**
*Fatigue*	Model 1	135.431	16	<0.001	0.210	0.178	0.243	0.786	0.772
	Model 2	26.871	15	0.030	0.057	0.000	0.091	0.984	0.984
*Daytime sleep*	Model 3	101.110	16	<0.001	0.175	0.143	0.208	0.829	0.818
	Model 4	28.514	15	0.019	0.063	0.000	0.097	0.975	0.975

Robust RSMEA, χ^2^, TLI and CFI are listed.

## Discussion

### Principal findings

We studied the role of self-reported cognitive and psychiatric symptoms at 3 months in predicting single-item measures of fatigue and daytime sleep 12 months after ischemic stroke. We found that patients who reported a decline in concentration and memory, as well as higher symptoms of anxiety and depression at 3 months were at a higher risk for *increased* fatigue and daytime sleep at 12 months. Also, self-reported mental distress (as defined by self-reported psychiatric and cognitive symptoms) at 3 months was found to predict fatigue and daytime sleep at 12 months, when also taking known confounders such as stroke severity, age, sex, and difficulties sleeping at night and their interrelations into account.

### Increased self-reported cognitive and psychiatric symptoms predict increased fatigue

Higher levels of anxiety and depression 3 months post-stroke were associated with increased fatigue at 12 months. This is in line with earlier studies that found a relationship of symptoms of anxiety and depression at 6 months with fatigue at 12 months after minor stroke (5).

Moreover, 2 additional interesting findings emerged. First, in our sample, approximately 20% of patients reported HADS-A and -D scores above cut-off, which is a somewhat lower prevalence than usually found during the first months post-stroke where around 36% of patients experience depression 2–6 months post-stroke (52) and around 24% report anxiety 1–5 months post-stroke (19). This may in part be explained by the rather mild stroke severity and patients with known depression and/or medication for depression prior to their stroke being excluded in our sample. Our results suggest that even relatively low levels of psychiatric symptoms may predict fatigue in the long-term. This substantiates prior findings which indicated that fatigue is related to depressive symptoms, also in stroke patients not meeting clinical criteria for a diagnosis of depression (22). Second, psychiatric symptoms were significantly associated with fatigue even when controlling for difficulties sleeping at night. While the cross-sectional associations between difficulties sleeping at night, psychiatric symptoms and fatigue are well established after ischemic stroke (53), our results enhance these findings by showing that symptoms of anxiety and depression at 3 months predict fatigue beyond difficulties with nighttime sleep at 12 months.

Aligning with earlier findings where self-reported mental slowness (26), and performance-based learning, memory, processing speed and attention (24) have been associated with fatigue after stroke in cross-sectional studies, we found that patients reporting a decline in concentration and memory at 3 months had an eight- and four-fold higher risk of increased fatigue at 12 months. Our results therefore substantiate and extend prior findings by highlighting that patients with self-reported concentration and memory difficulties in the subacute phase may be at a higher risk for increased fatigue in the long-term after stroke.

The results of our study indicate that even stroke patients with relatively few self-reported cognitive difficulties after stroke may develop fatigue at 12 months. With approximately one third of patients reporting a decline in concentration and memory in our sample, the prevalence of self-reported cognitive difficulties was rather low compared to other studies where 54% of patients reported concentration difficulties and 78% memory difficulties 3 months after mild ischemic stroke (54), and up to 92% reported cognitive difficulties during the first four and a half years after stroke (25). This may in part be due to different assessment methods of self-reported cognitive symptoms. In this study, we asked the patients to compare their memory and concentration 3 months after stroke to before the stroke. With no “gold standard” to assess self-reported cognitive symptoms post-stroke (25), and with many studies not including a measure for pre-stroke cognitive function, our study may therefore provide new insights regarding the role of self-reported *changes* in cognitive symptoms after stroke.

As of yet, there is insufficient evidence to support any effective prevention of post-stroke fatigue (55), but cognitive behavioral therapy (CBT) has shown promising affects in reducing fatigue compared to treatment as usual in a group of stroke patients (56), and compared to an health education intervention in a mixed sample of patients with stroke and traumatic brain injury (57). Besides targeting fatigue itself, CBT may also reduce self-reported cognitive and psychiatric symptoms, as well as sleep problems which then may have secondary benefits for preventing fatigue. Indeed, some evidence suggest that CBT decreased depressive symptoms in addition to fatigue, and increased self-reported sleep quality approximately 20 months post-stroke (56). As pharmacological interventions have not shown promising effects on managing post-stroke fatigue (55), despite successfully treating depression (58, 59), CBT may be more suitable in targeting the multifactorial nature of fatigue post-stroke.

### Increased self-reported cognitive and psychiatric symptoms may predict daytime sleep

Exploring the relationship between cognitive symptoms and daytime sleep, our findings indicate that a decline in concentration and memory may be associated with a three to five-fold risk of increased daytime sleep after stroke. This is in line with studies on the relationship between daytime sleep and performance-based cognitive function in the general elderly population which have shown that longer intervals of daytime sleep (≥ 120 min/day) increase the risk for developing cognitive impairment after 12 years by more than 60% (33). Also, patients with higher levels of psychiatric symptoms may be at a higher risk for increased daytime sleep. This is consistent with studies proposing depression as a possible predictor of future daytime sleepiness after stroke (30), and with findings indicating a higher need for sleep in patients with depression (60).

Yet, while some patients may sleep more during daytime because of psychiatric and cognitive symptoms, it may also be a conscious strategy to improve or prevent these symptoms. For example, engaging in daytime sleep may be an attempt to restore cognitive function and an indication of patients being able to adequately evaluate their needs. Medium duration intervals of daytime sleep (31–60 min) have shown to benefit delayed word recall in the general older population (35), but did not enhance behavioral function after motor training in stroke patients (61), and has been associated with less functional recovery during rehabilitation (62). Future research needs to further clarify whether engaging in daytime sleep may be an effective strategy to prevent self-reported cognitive decline after stroke.

### Mental distress predicts both fatigue and daytime sleep

We defined mental distress as a variable including self-reported symptoms of anxiety and depression, as well as self-reported decline in memory and concentration. Aligning with previous research that found a strong relationship between psychiatric symptoms and cognition, especially when self-reported measures were used (54) and with concentration difficulties being a diagnostic criterion for depression in the DSM-5 (23), worsened self-reported cognitive symptoms were significantly associated with clinical levels of anxiety and depression at 3 months in our sample. We therefore assume that these self-reported measures may capture a feeling of being mentally distressed. Indeed, screening tools such as HADS, have earlier been criticized as measures of general psychiatric distress rather than symptoms of anxiety and depression specifically (63).

Using SEM, we found that self-reported mental distress predicts fatigue and daytime sleep at 12 months. This may be valuable to identify patients at a higher risk for these symptoms. Given that mental distress can be self-reported and does not require extensive neuropsychological testing or trained staff, short self-report screening measures may be an easier to implement tool during standard clinical follow-up.

Also, self-reported mental distress predicted difficulties sleeping at night at 12 months to a similar degree as fatigue and daytime sleep. Symptoms of insomnia have been associated with higher levels of anxiety and depression (53, 64, 65), as well as higher suicidality 3 months post-stroke (66). With about half of stroke patients suffering from difficulties sleeping at night (67), this is a common stroke sequela, and identifying those patients with higher self-reported mental distress in the subacute phase may help to prevent sleep difficulties in the long-term.

Furthermore, difficulties sleeping at night has been found to predict daytime sleepiness after ischemic stroke (28), likely due to disturbance of the circadian rhythm (30), and have been associated with increased fatigue post-stroke (12). Thus, increased difficulties sleeping at night may also play a mediating role between mental distress at 3 months and increased fatigue and daytime sleep at 12 months.

In the SEM-models with mental distress included as predictor, lower age predicted increased fatigue, and higher age increased daytime sleep at 12 months. Higher age is commonly related to increased sleep during the daytime (68), and younger patients may experience increased fatigue post-stroke because they may be more actively involved in professional and/or social activities.

In our analyses, the χ^2^ tests were significant also for those models including mental distress as a predictor. The χ^2^ test has been criticized for being less informative for larger sample sizes by too easily rejecting appropriate fit to the data (69). Since it tests the null hypothesis that the SEM-model is perfectly specified, a significant test statistic for our models is unsurprising as they cannot be expected to include *all* factors that contribute to predicting fatigue and daytime sleep post-stroke.

### Strengths, limitations and future directions

This study extends prior research by focusing on temporal relationships of self-reported cognitive and psychiatric symptoms at 3 months with fatigue and daytime sleep at 12 months, and provides new insights that may be valuable both for research and clinical practice. Further, we used SEM to evaluate complete theoretically specified models, adding a higher-level perspective to the analyses (70), and may therefore better account for the complex nature of these post-stroke sequelae.

However, several other factors potentially contributing to fatigue and daytime sleep 12 months after stroke, such as medication, comorbidities, stroke recurrence, disability as well as fatigue and daytime sleep at 3 months were not included in this study. Such measures were either not available or had too many missing values to be included in analyses.

One major limitation of this study is the use of non-validated single-item self-reported measures to assess cognitive symptoms, fatigue, difficulties sleeping at night and daytime sleep which may not capture the complex nature of these sequelae. Such symptoms may have been underreported and interpreted differently by patients: For example, “difficulties sleeping at night” may cover difficulties with falling asleep, maintaining sleep and/or early morning awakening; similarly, self-reported decline in memory may reflect a perceived decline in a broader range of cognitive domains as patients with brain damage tend to interpret reduced processing speed, attention and executive control as memory deficits (71). Also, self-reported changes may be susceptible to recall bias, and it is difficult to know to which degree the single-item measures of fatigue and daytime sleep used in this study are measures of post-stroke fatigue and daytime sleep, or rather measures of general mental distress.

As little is known about the relationships between self-reported symptoms and daytime sleep post-stroke, these analyses were exploratory and were not statistically adjusted for multiple testing. Findings should therefore be interpreted with caution.

In this study, we aimed to identify possible predictors of fatigue and daytime sleep and focused on self-reported symptoms at 3 months. These relationships may be mediated by mental distress at 12 months, and thus, increased psychiatric symptoms may partially explain our findings. Also, fatigue and daytime sleep in the subacute phase may predict mental distress in the long-term.

While the majority of the Norwegian stroke population experience mild strokes (72), the analyzed sample had significantly milder strokes and higher MMSE-score compared to the rest of the NORSPOT cohort. Our findings may therefore be more generalizable to patients with relatively good outcomes after ischemic stroke. Finally, collecting data *via* postal questionnaires lead to missing data in several variables included which may bias results, and selection bias may not be ruled out as we only included patients who answered both the 3- and 12-month questionnaire.

Future studies should include validated measures, larger sample size and additional predictor variables to further study the predictive role of cognitive and psychiatric symptoms for fatigue and daytime sleep post-stroke.

## Conclusion and clinical implications

This study indicates that self-reported cognitive and psychiatric symptoms 3 months post-stroke may be powerful predictors of increased post-stroke fatigue and daytime sleep at 12 months—two stroke sequelae that may impair patients' daytime functioning and rehabilitation.

This may be especially bothersome and disabling for patients with mild stroke and relatively good outcome as in our sample, because such patients are more independent and involved in social and/or professional activities, while increased fatigue and daytime sleep may be experienced as rather minor stroke sequela in patients with severe neurological, cognitive or physical impairment (8). Thus, patients with minor stroke may especially benefit from interventions for cognitive and psychiatric symptoms that may have secondary benefits for preventing fatigue and daytime sleep in the long-term after stroke.

Therefore, an early self-report screening of psychiatric and/or cognitive difficulties, and a more extensive follow-up for those at risk may help to alleviate some of the symptoms and counter hidden impairment in these patients. Finally, increased psychoeducation for clinicians, patients, and their social network, may help to heighten awareness of how important it might be to detect and treat hidden psychiatric and cognitive symptoms after stroke.

## Data availability statement

The raw data supporting the conclusions of this article will be made available by the authors, without undue reservation.

## Ethics statement

The studies involving human participants were reviewed and approved by Privacy Ombudsman at Ahus (Approval number 11-076). Written informed consent for participation was not required for this study in accordance with the national legislation and the institutional requirements.

## Author contributions

EK developed the study design, conducted all analyses, interpreted and visualized results, and wrote the first and final draft of the manuscript. AL and MB contributed to the study design, data collection, interpretation of results, and critical appraisal of the manuscript. MB provided advice and feedback on all statistical analyses used in the paper and is the principal investigator of NORSPOT. AO and EG contributed to the study design, critical review, and editing of the manuscript. BT contributed to critical review and editing of the manuscript. OT contributed advice about modeling decisions within the SEM framework, concerning model specification, model fit indices, and implementation in the lavaan package and also provided feedback on the statistics used in the entire paper and the academic use of the English language. BI contributed to critical review of the manuscript. RG contributed to the conceptualization and planning of the study design and also to drafting, critical review, and editing of the manuscript. All authors approved the final version of the manuscript.

## Funding

MB and EK have been partially funded by NRC grants Simulation Simulating Patient Flow and Health Care Costs in Norway No. 196454 and Modelling Treatment and Rehabilitation of Stroke Patients - Using Simulation to Evaluate the Present and Plan for the Future No. 237809.

## Conflict of interest

The authors declare that the research was conducted in the absence of any commercial or financial relationships that could be construed as a potential conflict of interest.

## Publisher's note

All claims expressed in this article are solely those of the authors and do not necessarily represent those of their affiliated organizations, or those of the publisher, the editors and the reviewers. Any product that may be evaluated in this article, or claim that may be made by its manufacturer, is not guaranteed or endorsed by the publisher.
