# Supplementary material for: Self-reported cognitive and psychiatric symptoms at 3 months predict single-item measures of fatigue and daytime sleep 12 months after ischemic stroke
Source: Front Neurol. 2022 Nov 17;13:944586. doi: 10.3389/fneur.2022.944586 (PMC9712190; doi:10.3389/fneur.2022.944586)
Supplement: Supplementary file 1 [file Data_Sheet_1.docx]

**Supplementary Material**

Supplementary Table 1. *Comparison between the analyzed and the non-analyzed sample.*

|  | Patients included in the analyses^1^ (*n*=156) | Patients not included in the analyses^2^ (*n*=559) | t/χ^2^ | *df* | *p* | *d/*φ_c_ | Data avai-lable |
| --- | --- | --- | --- | --- | --- | --- | --- |
| Age in years, M (SD)^3^ | 73.00 (10.78) | 74.34 (14.41) | 1.27 | 325.61 | .21 | .10 | 715 |
| Sex, n female (%) | 41.00 | 46.30 | 1.39 | 1.00 | .24 | .04 | 715 |
| NIHSS score within 24 hours after admission, M (SD)^3^ | 3.59 (4.27) | 6.97 (7.29) | 6.78 | 402.89 | < .001* | .51 | 590 |
| MMSE during hospital stay (range 0-30), M (SD)^3^ | 25.34 (4.83) | 23.19 (5.77) | -3.31 | 195.75 | .001* | .39 | 301 |
| CDT during hospital stay (range 0-5), M (SD) | 3.71 (1.39) | 3.50 (1.51) | -1.10 | 291 | .28 | .14 | 293 |
| mRS day 7 (range 0-5), M (SD) | .99 (.09) | .99 (.09) | -.002 | 481 | > .99 | .00 | 483 |

*Note.* ^1^Patients who answered both the three- and 12-month questionnaire and met the inclusion criteria.^2^Patients where data at three- and/or 12-months was unavailable. ^3^Welch’s independent samples t-test was used due to unequal variances. *Significant group difference, p ≤ .05. MMSE=Mini-Mental State Examination, measuring general cognitive function. CDT=The Clock Drawing Test, assessing visuospatial and executive function. mRS= modified Rankin Scale, assessing dependence in activities of daily living and disability post-stroke. A mRS-score of six denotes death. mRS was assessed at the SU seven days after admission (or at discharge if sooner).

Supplementary Table 2. *Full regression model with HADS-A predicting fatigue.*

|  | | **Increased fatigue (12 months)** | | | | | | |  |
| --- | --- | --- | --- | --- | --- | --- | --- | --- | --- |
|  | | |  | 95% CI | |  |  | |  |
| **Predictors** | | | OR | Lower | Upper | | p | R^2^ |  |
|  | Age | | .94 | .89 | .98 | | .006* | .43 |  |
|  | Sex (female) | | 2.14 | .82 | 5.64 | | .12 |  |  |
|  | NIHSS | | .96 | .85 | 1.07 | | .45 |  |  |
|  | Difficulties sleeping at night (12 months) | | 9.21 | 2.24 | 37.96 | | .002* |  |  |
|  | HADS-A (3 months) | | 1.28 | 1.12 | 1.47 | | < .001* |  |  |

*Note:* * = Significant result at p ≤ .0125. R^2^=Nagelkerke R^2^

Supplementary Table 3. *Full regression model with HADS-D predicting fatigue.*

|  | | **Increased fatigue (12 months)** | | | | | | |  |
| --- | --- | --- | --- | --- | --- | --- | --- | --- | --- |
|  | | |  | 95% CI | |  |  | |  |
| **Predictors** | | | OR | Lower | Upper | | p | R^2^ |  |
|  | Age | | .93 | .89 | .98 | | .003* | .39 |  |
|  | Sex (female) | | 2.41 | .92 | 6.31 | | .07 |  |  |
|  | NIHSS | | .95 | .84 | 1.07 | | .37 |  |  |
|  | Difficulties sleeping at night (12 months) | | 8.81 | 2.22 | 34.96 | | .002* |  |  |
|  | HADS-D (3 months) | | 1.22 | 1.08 | 1.37 | | .001* |  |  |

*Note:* * = Significant result at p ≤ .0125. R^2^=Nagelkerke R^2^

Supplementary Table 4. *Full regression model with concentration predicting fatigue.*

|  | | **Increased fatigue (12 months)** | | | | | | |  |
| --- | --- | --- | --- | --- | --- | --- | --- | --- | --- |
|  | | |  | 95% CI | |  |  | |  |
| **Predictors** | | | OR | Lower | Upper | | p | R^2^ |  |
|  | Age | | .93 | .89 | .98 | | .007* | .42 |  |
|  | Sex (female) | | 2.68 | .96 | 7.45 | | .06 |  |  |
|  | NIHSS | | .96 | .84 | 1.08 | | .50 |  |  |
|  | Difficulties sleeping at night (12 months) | | 15.01 | 3.37 | 66.79 | | < .001* |  |  |
|  | Worse concentration (3 months) | | 7.68 | 2.35 | 25.04 | | < .001* | . |  |

*Note:* * = Significant result at p ≤ .0125. R^2^=Nagelkerke R^2^

Supplementary Table 5. *Full regression model with memory predicting fatigue.*

|  | | **Increased fatigue (12 months)** | | | | | | |  |
| --- | --- | --- | --- | --- | --- | --- | --- | --- | --- |
|  | | |  | 95% CI | |  |  | |  |
| **Predictors** | | | OR | Lower | Upper | | p | R^2^ |  |
|  | Age | | .93 | .89 | .98 | | .004* | .40 |  |
|  | Sex | | 3.19 | 1.14 | 8.89 | | .03 |  |  |
|  | NIHSS | | .97 | .86 | 1.09 | | .58 |  |  |
|  | Difficulties sleeping at night (12 months) | | 10.97 | 2.68 | 44.91 | | < .001* |  |  |
|  | Worse memory (3 months) | | 4.05 | 1.48 | 11.10 | | .007* |  |  |

*Note:* * = Significant result at p ≤ .0125; R^2^=Nagelkerke R^2^

Supplementary Table 6. *Full regression model with HADS-A predicting daytime sleep.*

|  | | **Increased daytime sleep (12 months)** | | | | | | |  |
| --- | --- | --- | --- | --- | --- | --- | --- | --- | --- |
|  | | |  | 95% CI | |  |  | |  |
| **Predictors** | | | OR | Lower | Upper | | R^2^ | |  |
|  | Age | | 1.02 | .98 | 1.05 | | .13 | |  |
|  | Sex (female) | | .59 | .27 | 1.29 | |  |  |  |
|  | NIHSS | | 1.01 | .93 | 1.10 | |  |  |  |
|  | Difficulties sleeping at night (12 months) | | 2.89 | 1.10 | 7.60 | |  |  |  |
|  | HADS-A (3 months) | | 1.11 | 1.01 | 1.24 | |  |  |  |

*Note:* R^2^=Nagelkerke R^2^

Supplementary Table 7. *Full regression model with HADS-D predicting daytime sleep.*

|  | | **Increased daytime sleep (12 months)** | | | | | | |  |
| --- | --- | --- | --- | --- | --- | --- | --- | --- | --- |
|  | | |  | 95% CI | |  |  | |  |
| **Predictors** | | | OR | Lower | Upper | | R^2^ | |  |
|  | Age | | 1.01 | .97 | 1.05 | | .13 | |  |
|  | Sex (female) | | .66 | .30 | 1.43 | |  |  |  |
|  | NIHSS | | 1.00 | .92 | 1.09 | |  |  |  |
|  | Difficulties sleeping at night (12 months) | | 2.96 | 1.13 | 7.73 | |  |  |  |
|  | HADS-D (3 months) | | 1.11 | 1.01 | 1.22 | |  |  |  |

*Note:* R^2^=Nagelkerke R^2^

Supplementary Table 8. *Full regression model with concentration predicting daytime sleep.*

|  | | **Increased daytime sleep (12 months)** | | | | | | |  |
| --- | --- | --- | --- | --- | --- | --- | --- | --- | --- |
|  | | |  | 95% CI | |  |  | |  |
| **Predictors** | | | OR | Lower | Upper | | R^2^ | |  |
|  | Age | | 1.03 | .99 | 1.08 | | .22 | |  |
|  | Sex (female) | | .42 | .17 | 1.03 | |  |  |  |
|  | NIHSS | | 1.03 | .92 | 1.15 | |  |  |  |
|  | Difficulties sleeping at night (12 months) | | 3.43 | 1.24 | 9.47 | |  |  |  |
|  | Worse concentration (3 months) | | 4.89 | 1.85 | 12.93 | |  |  |  |

*Note:* R^2^=Nagelkerke R^2^

Supplementary Table 9. *Full regression model with memory predicting daytime sleep.*

|  | | **Increased daytime sleep (12 months)** | | | | | | |  |
| --- | --- | --- | --- | --- | --- | --- | --- | --- | --- |
|  | | |  | 95% CI | |  |  | |  |
| **Predictors** | | | OR | Lower | Upper | | R^2^ | |  |
|  | Age | | 1.02 | .98 | 1.06 | | .19 | |  |
|  | Sex (female) | | .60 | .26 | 1.38 | |  |  |  |
|  | NIHSS | | 1.00 | .91 | 1.09 | |  |  |  |
|  | Difficulties sleeping at night (12 months) | | 3.31 | 1.20 | 9.12 | |  |  |  |
|  | Worse memory (3 months) | | 3.39 | 1.45 | 7.94 | |  |  |  |

*Note:* R^2^=Nagelkerke R^2^
